# Farm animals for studying muscle development and metabolism: dual purposes for animal production and human health

**DOI:** 10.1093/af/vfz015

**Published:** 2019-06-25

**Authors:** Liang Zhao, Yan Huang, Min Du

**Affiliations:** 1 Department of Animal Sciences, Washington State University, Pullman, WA; 2 Department of Animal Science, University of Arkansas, Fayetteville, AR

**Keywords:** development, farm animals, human study, metabolism, skeletal muscle

**Implications**

- Nutritional fluctuation during the fetal stage alters skeletal muscle development, which has long-term effects on offspring.
- Dysregulation of satellite cells and fibro/adipogenic progenitors leads to muscle atrophy, fatty infiltration, and fibrosis in humans, while enhancing fibro/adipogenic proliferation may increase marbling in meat animals.
- Muscle fiber composition affects animal growth efficiency and meat quality, while in humans, shifting oxidative to glycolytic myofibers is associated with metabolic syndromes.
- Thermogenesis in the skeletal muscle and brown adipose tissue elevates energy expenditure and heat production, which prevents obesity and metabolic diseases in humans but may reduce feed efficiency in animals.
- The long duration of pregnancy and lactation of farm animals provides unique opportunities for stage-specific nutrient interventions to precisely manage and optimize muscle growth and carcass composition.

## Introduction

In animal production, skeletal muscle provides meat for human consumption. Skeletal muscle is mainly comprised of muscle fibers, connective tissues, and intramuscular adipose tissues. The characteristics of muscle fibers and connective tissues determine meat tenderness, whereas intramuscular adipose content (marbling fat) is the major factor determining the flavor and juiciness of meat (Hocquette et al., 1998). Since myocytes, adipocytes, and fibroblasts are all derived from the same pool of mesenchymal stem/progenitor cells in dermomyotome during early embryonic development, their commitments to different lineages can be considered as a competitive process (Du et al., 2015). Nutritional intervention which enhances myogenesis and intramuscular adipogenesis while suppressing fibrogenesis may improve animal production efficiency and meat quality.

In humans, ectopic lipid accumulation in skeletal muscle induces local inflammation and insulin resistance, leading to development of metabolic dysfunction and diabetes mellitus (Akhmedov and Berdeaux, 2013). In addition, excessive fibrotic tissue formation is commonly seen in dystrophic muscle (Mahdy, 2018). Fibrosis disrupts muscle homeostasis, weakens muscle contraction force, and impairs its metabolic function. Satellite cells and fibro/adipogenic progenitors are two groups of progenitor cells in adult skeletal muscle responsible for muscle fiber regeneration and growth (Wosczyna and Rando, 2018). However, dysregulation of these progenitor cells is correlated with muscle atrophy, fatty infiltration, and fibrosis.

Energy metabolism in skeletal muscle is precisely regulated to meet the energy requirements for physiological functions such as growth, physical activity, and thermogenesis (Hocquette et al., 1998). Metabolic studies in farm animals have focused on increasing muscle growth efficiency and meat quality, while decreasing production costs. On the other hand, enhancing energy expenditure in skeletal muscle contributes greatly to the maintenance of whole-body energy homeostasis and metabolic health. Though ruminants and humans exhibit different characteristics in carbohydrate and lipid metabolism, they share similar regulatory mechanisms. Understanding the roles of skeletal muscle and adipose tissue in the core temperature maintenance and body weight control can help us to improve muscle growth and alleviate metabolic disorders. Collectively, studies to understand the biological processes of skeletal muscle development and metabolism in farm animals will not only benefit animal and meat production, but also human health.

## Skeletal Muscle Development

When tissues or organs are actively developing, they are susceptible to nutrient fluctuations. Fetal programming, also called developmental programming, describes the response of organisms to challenges during a critical developmental time window that alters the trajectory of development qualitatively and/or quantitatively with resulting persistent effects (Barker, 1997). Improper maternal nutrition impairs fetal development, which affects the long-term growth performance and health of offspring. Skeletal muscle is a major tissue for energy utilization and maintenance of metabolic health in humans, while providing lean tissues for meat animals. Thus, understanding the intrinsic regulatory mechanisms and timeline of skeletal muscle development during early life is essential for improving animal growth and the efficiency of meat production as well as improving human health.

### Myogenesis

**Figure 1.**
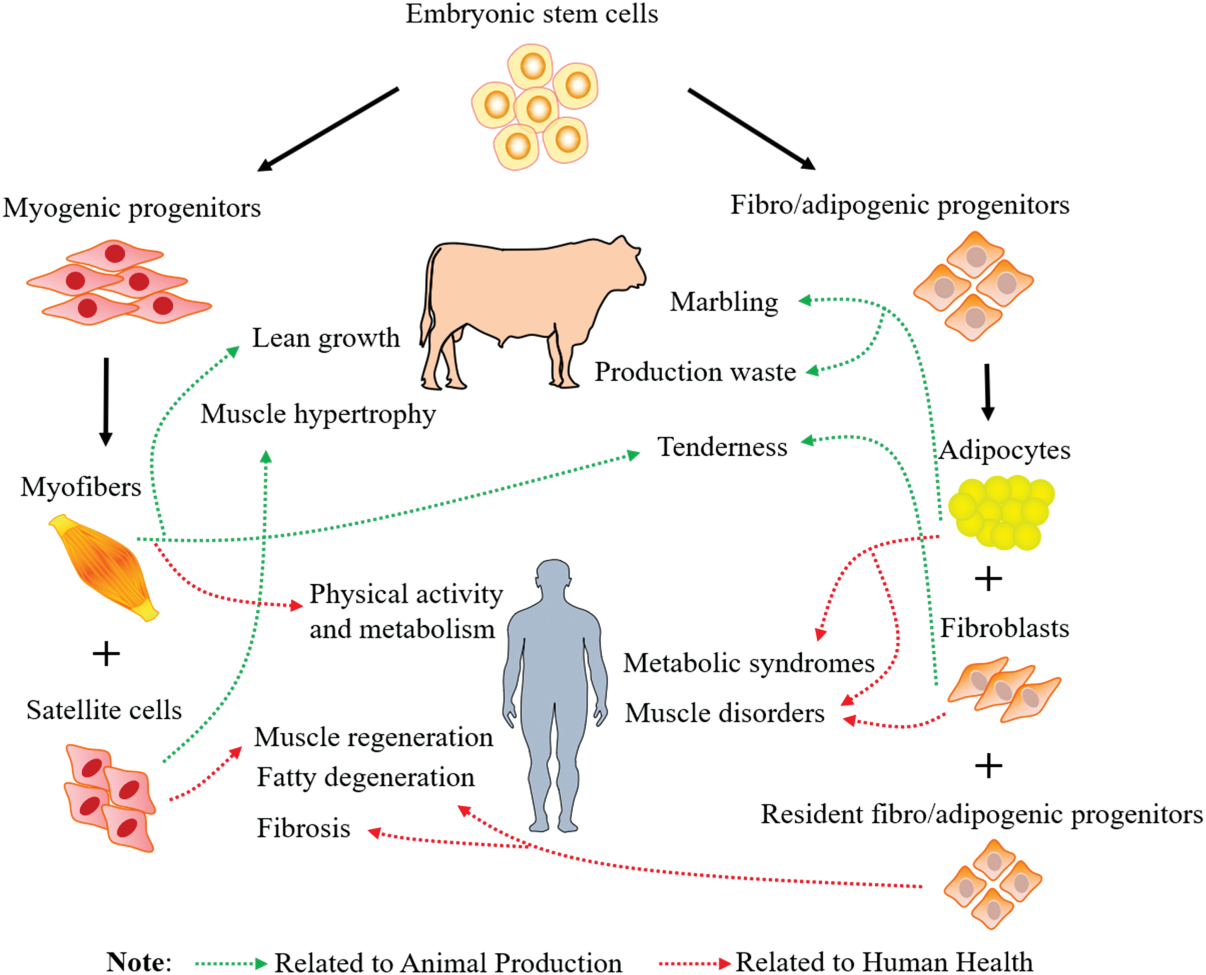
Mesenchymal progenitor cells differentiate into myogenic and fibro-adipogenic cells during fetal muscle development in humans and beef cattle. Impacts of early muscle development on animal production and human health are indicated. Marbling is another term for intramuscular fat in skeletal muscle (e.g., meat).

**Figure 2.**
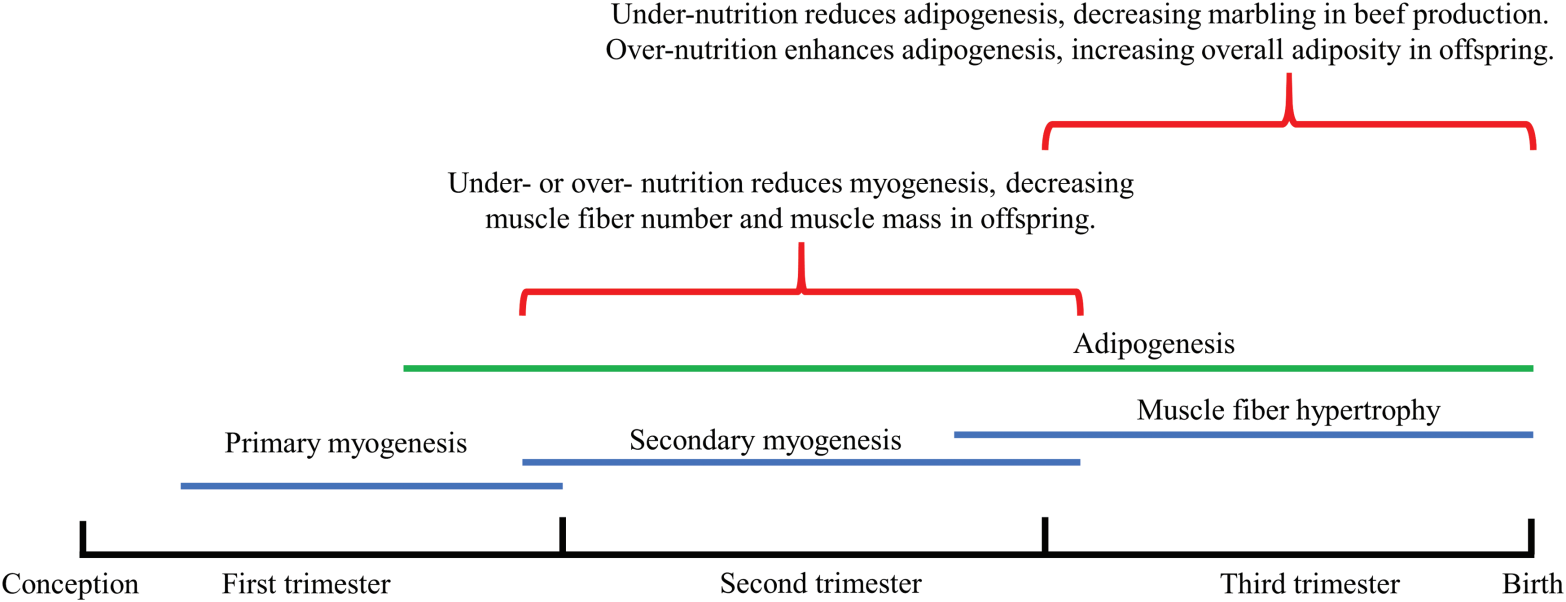
Effects of maternal nutrition on fetal skeletal muscle and adipose tissue development of both beef cattle and humans. The timelines are estimated.

Prenatal muscle development can be separated into embryonic and fetal stages (Du et al., 2010a). Consequently, muscle development can be divided into primary and secondary myogenesis, which occurs primarily during the embryonic and fetal stages, respectively. During embryonic development, mesenchymal stem cells in myotome commit to the myogenic lineage through the expression of transcription factors including paired box gene (Pax) 3 and Pax7 (Buckingham et al., 2003). These committed myoblasts proliferate and fuse to form primary muscle fibers under the control of myogenic regulatory factors (MRFs), including Myf-5, Myo-D, myogenin, and MRF-4 (Buckingham et al., 2003). Although a very limited number of muscle fibers are formed in this stage, these primary muscle fibers serve as templates for the formation of secondary muscle fibers during the fetal stage (Swatland, 1973). Myogenic precursor cells surrounding primary muscle fibers profoundly proliferate to increase their numbers and then fuse to form secondary muscle fibers (Beermann et al., 1978). Most muscle fibers in adults are formed during the secondary myogenesis. During the late fetal and neonatal stages, a portion of myogenic cells become quiescent to form satellite cells (Figure 1). Thus, the population size of myogenic precursor cells not only determines the number of muscle fibers formed but also affects the density of satellite cells in postnatal muscle. Because the number of muscle fibers does not increase after birth, myogenesis during the fetal stage has profound impacts on production efficiency of animals, as well as human health (Du et al., 2010a, 2010b) (Figure 2).

Satellite cells fuse with the preexisting muscle fibers and contribute to the postnatal muscle growth in size (muscle hypertrophy) (Figure 1). In adult muscle, satellite cells are in a quiescent state and become activated in response to external stimuli such as exercise and injury. Activated satellite cells proliferate, differentiate, and fuse with preexisting muscle fibers to repair damaged muscle fibers or regenerate new fibers in injured muscle (Figure 1). Decreased density and disrupted function of satellite cells are associated with impaired regenerative capacity and muscle degeneration due to diseases or disorders (Fukada, 2018).

### Adipogenesis and Fibrogenesis

Recent studies show that adipocytes and fibroblasts are derived from a common source of progenitor cells, called fibro/adipogenic progenitors (Joe et al., 2010; Uezumi et al., 2010). A large population of fibro/adipogenic progenitors form during the fetal stage, which develop into adipocytes, whereas a portion reside surrounding the vascular system to become resident fibro/adipogenic progenitors in mature adipose tissue (Jiang et al., 2014; Du et al., 2015) (Figure 1). The resident fibro/adipogenic progenitors are responsible for adipose homeostasis in adult animals (Jiang et al., 2014). The early adipogenic commitment overlaps with the period of secondary myogenesis (before the second trimester for beef cattle), and most adipocytes are formed during late gestation to the neonatal stage (Du et al., 2010a). It is reported that the total number of adipocytes is set before adolescence in humans (Spalding et al., 2008). Therefore, regulation of the formation of adipogenic precursors during the early developmental stage affects adipocyte hyperplasia and the overall mass of adipose tissue.

**Figure 3.**
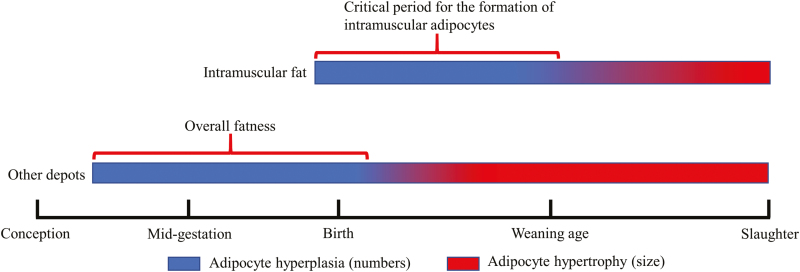
Chronological difference of adipogenesis in different depots of beef cattle.

Development of visceral, subcutaneous, intermuscular, and intramuscular adipose tissue follows a sequential order in livestock. Visceral and subcutaneous adipose tissues develop first, initiate at the end of the first trimester in ruminants and before mid-gestation in rodents (Jiang et al., 2014). Formation of intramuscular adipocytes mainly occurs during the late fetal to about 250 d of age in beef cattle (Du et al., 2015) (Figure 3). The chronological difference in development of adipose depots and a long duration of gestation provides an opportunity to increase intramuscular fat deposition while minimizing production costs (Figure 3). Accompanied with adipogenesis, fibrogenesis is also active during the fetal stage and generates connective tissue which forms primordial endomysium, perimysium, and epimysium in skeletal muscle during late gestation. Fibroblasts also produce enzymes to catalyze collagen cross-linking which contributes to the background toughness of meat. Thus, reducing fibrogenesis during muscle development contributes to the reduction of meat toughness (Du et al., 2013).

In humans, a portion of fibro/adipogenic progenitors are located in the interstitial region between muscle fibers, which is responsible for fatty infiltration and fibrosis production in diseased or disordered skeletal muscle (Joe et al., 2010). In addition, in a model of muscle injury, fibro/adipogenic progenitors are activated earlier and secrete cytokines to promote the function of satellite cells for muscle regeneration (Joe et al., 2010). However, over-proliferation of fibro/adipogenic progenitors causes fatty degeneration and fibrosis in the regenerated muscle which impairs muscle repair (Figure 1). Therefore, understanding the mechanisms that regulate development of satellite cells and fibro/adipogenic progenitors will help develop therapies for muscle disease or disorders.

### Nutritional intervention of myogenesis, adipogenesis, and fibrogenesis

Compared with brain and heart tissues, skeletal muscle has a lower priority for nutrient repartitioning, which makes the development of skeletal muscle especially vulnerable to nutritional changes. As myogenesis, adipogenesis, and fibrogenesis mainly occur during the fetal stage, nutritional regulation of their proliferation and differentiation has long-term effects on the composition and performance of skeletal muscle in offspring (Figure 2). In sheep, our previous studies showed that both maternal under- and over-nutrition during mid-gestation decreased myogenesis during fetal development, but only over-nutrition increased intramuscular fat deposition. In contrast, under-nutrition followed by over-nourishment after birth led to overall adiposity in offspring (Zhu et al., 2006; Zhu et al., 2008). Furthermore, increased fibrogenesis was found in the skeletal muscle of lambs born from the ewes treated with maternal over-nutrition during gestation (Huang et al., 2010).

Since adipocytes and fibroblasts are derived from bipotent fibro/adipogenic progenitors, manipulation of the lineage commitment of fibro/adipogenic progenitors provides an opportunity to enhance intramuscular adipogenesis while decreasing fibrogenesis. However, the mechanisms determining adipogenic or fibrogenic differentiation of fibro/adipogenic progenitors and their maintenance in skeletal muscle remain poorly understood.

## Metabolism of Skeletal Muscle

Skeletal muscle mainly uses carbohydrate and lipids for energy production to support various biological functions, including growth, physical activity, and thermogenesis. The partition of energy substrates between muscle, intramuscular fat, and subcutaneous fat not only affects muscle growth efficiency and meat quality, but also has systematic effects on whole-body metabolism. Skeletal muscle accounts for a large portion of resting metabolic rate and energy consumption, which may be critical for core temperature maintenance and body weight control in response to external stimuli. Studies on the mechanisms regulating glucose and lipid metabolism and thermogenesis in skeletal muscle will help us to improve animal growth and meat production efficiency, as well as human health.

### Muscle fiber composition and growth

According to the contractile and metabolic properties, skeletal muscle can be roughly classified as slow-twitch oxidative (type Ⅰ) fibers, fast twitch oxidative-glycolytic fibers (type Ⅱa), and fast-twitch glycolytic fibers (type Ⅱb) in rodents and pigs. In large mammals including humans and ruminants, type IIx replaces type IIb as the dominant fast-twitch fibers. The relative proportions of myofibers with different metabolic properties are determined by genetic factors and influenced by physiological, hormonal, and nutritional factors (Hocquette et al., 1998). Genetic selection of animals with rapid growth rates and leanness is associated with a higher proportion of glycolytic myofibers (Hocquette, 2010). Since glycogen content determines ultimate postmortem pH which affects meat quality, a higher proportion of glycolytic myofibers reduces ultimate pH and water holding capacity of meat.

Ectopic lipid accumulation can be separated into intramuscular and intramyocellular deposition. Because meat animals are typically harvested at a young age, the amount of intramyocellular lipid is limited and correlates with the presence of type I fibers, but in humans, intramyocellular and to a lesser extent intramuscular lipid accumulation leads to insulin resistance. Ectopic lipids in skeletal muscle induce local inflammation and impair downstream insulin signaling, which disrupts glucose metabolism and leads to development of metabolic dysfunction (Akhmedov and Berdeaux, 2013). Under insulin resistance states such as obesity, diabetes, aging, and physical inactivity, skeletal muscle showed reduced oxidative capacity and increased glycolytic capacity (Gaster et al., 2001). Therefore, oxidative and glycolytic metabolism in skeletal muscle are tightly regulated to optimize energy expenditure, and glycogen and fat storage to support various biological functions. The flexibility in glycolytic and oxidative capacities of skeletal muscle reflects the adaption of skeletal muscle to meet the requirements of specific biological functions such as muscle growth or pathophysiological conditions.

### Carbohydrate and lipid metabolism

It should be noted that ruminants and humans exhibit different characteristics in terms of carbohydrate and lipid metabolism. In contrast to humans and nonruminants, which absorb monosaccharides in the small intestine, ruminants absorb short-chain fatty acids due to the extensive rumen fermentation of carbohydrates (Huntington, 1997). Consequently, nonruminants and humans use glucose and long-chain fatty acids as the principal sources of energy, whereas ruminants mainly utilize volatile fatty acids. Nonetheless, glucose is still indispensable for the growth and development of ruminants, which is mainly provided through hepatic gluconeogenesis (Nafikov and Beitz, 2007). In terms of de novo lipogenesis, liver is the principal site for fatty acid synthesis in humans and rodents while lipogenesis happens primarily in adipose tissue of ruminants and swine. In addition, glucose is used for de novo lipogenesis in humans while ruminants mainly use rumen-derived acetate. Interestingly, in beef cattle, lipogenesis in the intramuscular fat prefers glucose rather than acetate as the primary substrate and diets rich in starch increased the deposition of intramuscular fat relative to subcutaneous fat (Choat et al., 2003), which may be due to differences between these two fat depots in the composition of fibro/adipogenic progenitors, preadipocytes, and mature adipocytes. Intramuscular fat develops later, is less mature, and glucose is required for producing glycerol and other intermediates for adipocyte maturation, whereas subcutaneous fat develops earlier, and acetate is preferred for the synthesis of fatty acid hydrocarbon chains. Understanding the similarities and differences in the regulatory mechanisms of carbohydrate and lipid metabolism utilized by ruminant animals and humans is essential for developing specific strategies to optimize animal production and human metabolic health.

### Thermogenesis in the skeletal muscle

**Figure 4.**
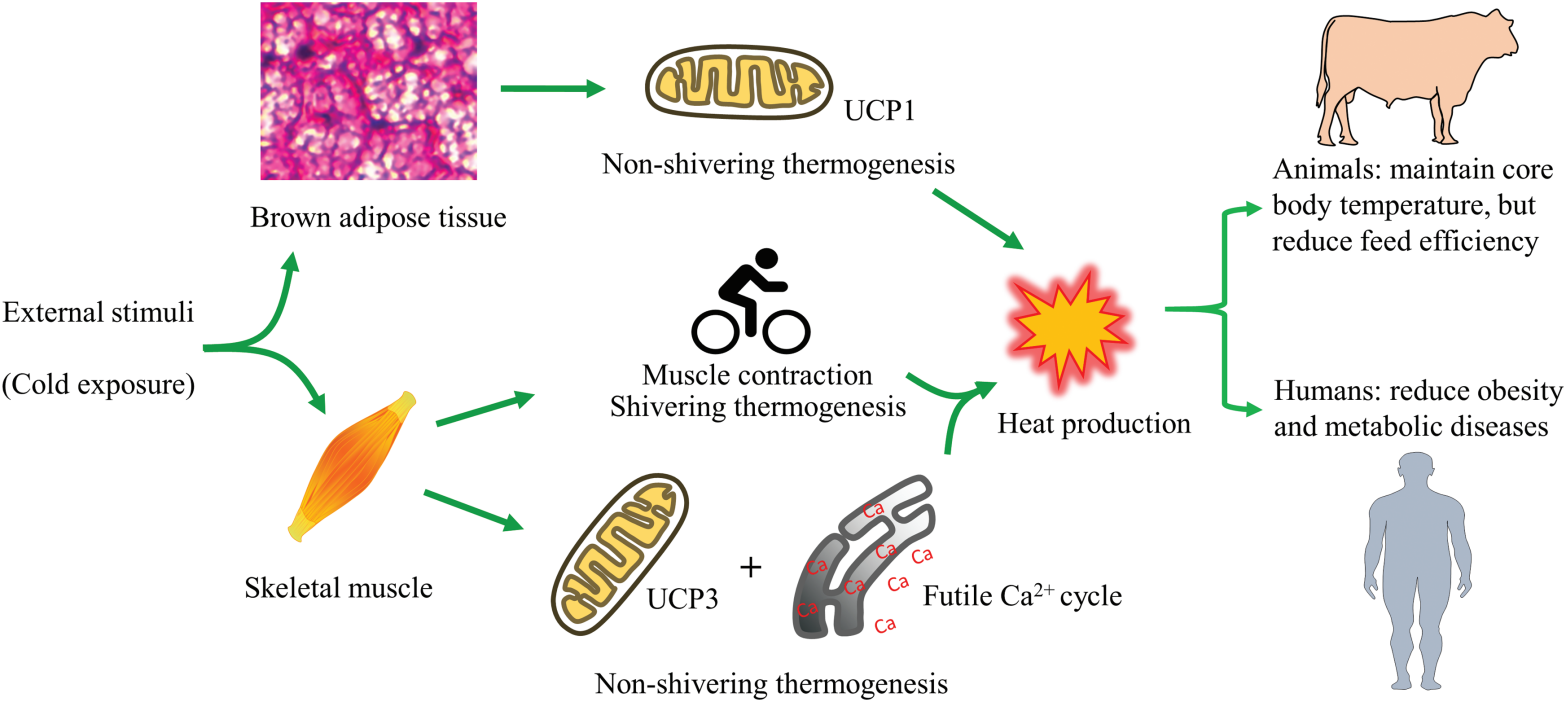
Thermogenesis from brown adipose tissue and skeletal muscle contribute to core temperature maintenance and body weight control in response to external stimuli such as cold exposure. (UCPs = uncoupling proteins).

Body temperature homeostasis is maintained by heat production which relies on both shivering and nonshivering thermogenesis (Figure 4). In response to acute cold exposure, shivering thermogenesis is activated immediately in the skeletal muscle to produce heat. In addition, brown adipose tissue is a primary site for nonshivering thermogenesis, which does not involve muscle contraction and relies on tissue-specific uncoupling protein 1 for heat generation. Uncoupling protein 1 decreases the proton gradient created in oxidative phosphorylation by driving protons across the inner membrane of mitochondria, preventing ATP synthesis and resulting in energy dissipation through heat production. Activation of uncoupling protein 1 is important for whole body energy expenditure and the prevention of obesity. In rodents, brown adipose tissue in the interscapular region remains throughout their lifespan. However, in humans and ruminants, brown adipose tissue is only abundant in neonates, and brown adipose tissue is replaced by white adipose tissue (Symonds, 2013). Recently, however, uncoupling protein 1-positive brown-like adipocytes were found in humans (Cypess et al., 2009) and also in intramuscular fat in beef cattle, which was interspersed amongst white adipose tissue depots of adults (Wei et al., 2018). These brown-like adipocytes are termed beige adipocytes, which are inducible and have been extensively studied in humans and rodents. However, the physiological contribution of brown-like adipocytes to energy homeostasis in farm animals remains largely unexplored.

Nonshivering thermogenesis was also found in skeletal muscle, where uncoupling protein 3 facilitates metabolic adaption and lipid metabolism and may function through a different mechanism from uncoupling protein 1 (Boss et al., 1997) (Figure 4). Futile calcium cycling between sarcoendoplasmic reticulum is an important mechanism of nonshivering thermogenesis in muscle (Clarke et al., 2012). Sarcolipin is an endogenous activator of sarcoendoplasmic reticulum ATPase, which transports calcium back into the sarcoendoplasmic reticulum and generates heat. Increased expression of sarcolipin in skeletal muscle has been associated with cold-induced thermogenesis and resistance of mice to the challenge of a high-fat diet (Bal et al., 2012). However, the role(s) that uncoupling protein 3 and sarcolipin play in thermogenesis of skeletal muscle in large mammals including humans and ruminants remain largely unexplored and requires further study. Since skeletal muscle accounts for approximately 40% of total body mass in large mammals, a small disturbance in muscle thermogenesis may contribute substantially to the total energy expenditure of the whole body, especially in adult animals when the contribution from nonshivering thermogenesis of brown adipose tissue is negligible. The body size of sheep is similar to humans and can be easily influenced by dietary management, making sheep a convenient model for studying body weight control. Therefore, ruminants, especially sheep, provide excellent models to delineate the role of nonshivering thermogenesis of skeletal muscle in maintaining body temperature and weight control (Figure 4).

## Conclusions

Although rodents have been widely used as models for studying human pathobiology, differences in body size, life span, and some physiological characteristics make rodents inappropriate biomedical models for many disease conditions affecting humans. Farm animals are much closer in body size to humans and share similar developmental patterns of skeletal muscle and adipose tissue, making them advantageous models for studying pathophysiological conditions in humans. Excessive intramuscular lipid accumulation in skeletal muscle is associated with impaired glucose metabolism and development of obesity and diabetes in humans, while improving meat quality in animals. However, up to now, our understanding of the origins and mechanisms that regulate intramuscular adipogenesis remain rudimentary. Farm animals can be easily sampled by muscle biopsies at different stages of development and can serve as excellent models for studying intramuscular adipogenesis. In addition, growth characteristics and intramuscular fat content vary greatly across different breeds of cattle. For example, Wagyu cattle have very high marbling (intramuscular fat), along with low subcutaneous fat thickness, which may be even lower than the subcutaneous fat thickness seen in Angus and other breeds of cattle commonly used for meat production. Moreover, compared with other common cattle breeds, Wagyu and Angus have similar growth characteristics. Due to similar growth rates but large differences in marbling, Wagyu and Angus are valuable models for studying preferential intramuscular adipogenesis and fat deposition with benefits to both animal production and human health.

Fetal programming at the prenatal stage determines skeletal muscle development and has long-term effects on offspring. Nutritional regulation at the fetal stage can alter the composition of muscle fibers, and the lean:fat ratio, which could benefit both animal production and human health. Due to the similarity in size, gestation length, and maternal burden, sheep are commonly used as models for fetal programming studies, even though the gestation length of sheep is only about half that of humans. Because muscle and adipose tissues develop during specific stages of gestation (Figures 2 and 3), the long duration of gestation and lactation in ruminants (especially beef cattle) allows stage-specific nutritional interventions to enhance animal growth performance and meat quality.

Finally, skeletal muscle plays a significant role in core temperature homeostasis and body weight control. Understanding the contribution of skeletal muscle to whole-body energy expenditure and heat production may help identify new treatments for obesity and associated metabolic syndromes in humans, and also increase feed efficiency in farm animals, especially beef cattle and sheep.

## Funding

This material is based upon work that is supported by the National Institute of Food and Agriculture, U.S. Department of Agriculture, under award numbers 2015-67015-23219 and 2016-68006-24634.

## About the Authors

**Liang Zhao** is a PhD student in Growth Biology in the Department of Animal Sciences at Washington State University. His research focuses on the physiology and metabolism in skeletal muscle with metabolic disorders. He originally came from China, with a BS in Food Quality and Safety, and a MS in Meat Science.

**Dr. Yan Huang** is an Assistant Professor in the Department of Animal Science, University of Arkansas. His research interests are nutritional regulation of muscle growth and development including myo-, adipo-, and fibrogenesis. With research experience in animal science and cell and molecular biology, he developed his independent research program at the University of Arkansas to focus on the function of mitochondria in muscle growth and meat quality.

**Dr. Min Du** is a Professor and Endowed Chair in the Department of Animal Sciences at Washington State University (WSU). He obtained his PhD from Iowa State University. Before joining WSU, he was a faculty member in the Department of Animal Science at the University of Wyoming. His current research focuses on the molecular mechanisms regulating mesenchymal progenitor cell differentiation, and early skeletal muscle and adipose tissue development, with applications to both human health and animal production.
